# YTHDC1-mediated microRNA maturation is essential for hematopoietic stem cells maintenance

**DOI:** 10.1038/s41420-024-02203-z

**Published:** 2024-10-16

**Authors:** Hongna Zuo, Jin Liu, Bin Shen, Yue Sheng, Zhenyu Ju, Hu Wang

**Affiliations:** 1 Department of Hematology, the Second Xiangya Hospital, Central South University, Changsha, Hunan China; 2 Zhejiang Key Laboratory of Medical Epigenetics, School of Basic Medical Sciences, The Third People’s Hospital of Deqing, Department of Cardiology, Affiliated Hospital of Hangzhou Normal University, Hangzhou Normal University, Hangzhou, China; 3 Department of Histology and Embryology, State Key Laboratory of Reproductive Medicine, Nanjing Medical University, Nanjing, Jiangsu China; 4 MOE Key Laboratory of Regenerative Medicine, Institute of Aging and Regenerative Medicine, Jinan University, Guangzhou, China

**Keywords:** Haematopoietic stem cells, Stem-cell research

## Abstract

YTHDC1, a reader of N6-methyladenosine (m^6^A) modifications on RNA, is posited to exert significant influence over RNA metabolism. Despite its recognized importance, the precise function and underlying mechanisms of YTHDC1 in the preservation of normal hematopoietic stem cell (HSCs) homeostasis remain elusive. Here, we investigated the role of YTHDC1 in normal hematopoiesis and HSCs maintenance in vivo. Utilizing conditional *Ythdc1* knockout mice and *Ythdc1/Mettl3* double knockout mice, we demonstrated that YTHDC1 is required for HSCs maintenance and self-renewal by regulating microRNA maturation. YTHDC1 deficiency resulted in HSCs apoptosis. Furthermore, we uncovered that YTHDC1 interacts with HP1BP3, a nuclear RNA binding protein involved in microRNA maturation. Deletion of YTHDC1 brought about significant alterations in microRNA levels. However, over-expression of *mir-125b*, *mir-99b*, and *let-7e* partially rescued the functional defect of YTHDC1-null HSCs. Taken together, these findings indicated that the nuclear protein YTHDC1-HP1BP3-microRNA maturation axis is essential for the long-term maintenance of HSCs.

## Introduction

Hematopoietic stem cells (HSCs) are the cornerstone of lifelong hematopoiesis, ensuring the continuous production of blood cells [1]. Transplantation of HSCs has demonstrated therapeutic potential in regenerative medicine, as well as in the treatment of hematologic malignancies and inherited blood disorders [2]. The enduring presence of HSCs throughout an individual’s lifespan is attributed to their capacity for differentiation and self-renewal [3]. While extensive research has begun to elucidate the molecular mechanisms that govern HSC function [4–14], a comprehensive understanding of the mechanisms underlying their long-term ex vivo maintenance remains elusive.

To date, over 150 distinct chemical modifications have been characterized within RNA molecules [15]. Prominent among these is N6-methyladenosine (m^6^A), which stands as a ubiquitous post-transcriptional modification in eukaryotic mRNA and non-coding RNAs. The functional implications of m^6^A are mediated by a cohort of RNA-binding proteins adept at ‘reading’ the m^6^A mark. These include YTHDF1, YTHDF2, YTHDF3, YTHDC2, eIF3, ELVAL, IGF2BP1, IGF2BP2, IGF2BP3, HNRNPA2B1, and PRRC2A, which collectively influence mRNA stability and translational efficiency [16, 17].

The N6-methyladenosine RNA modification is essential for the proper function and maintenance of HSCs. The m^6^A methyltransferases METTL3 and METTL14, which serve as ‘writers’ of this modification, are indispensable for HSCs homeostasis [18–21]. Notably, some studies have demonstrated that the absence of *Mettl3* or the m^6^A reader *Ythdf2*, but not *Mettl14*, leads to an increase in the number of HSCs [18–20, 22, 23]. Research conducted in Linheng Li’s laboratory and our own has revealed that *Ythdf2* is critically involved in the maintenance and stress response mechanisms of HSCs, primarily through its regulation of mRNA decay. This regulation affects the transcripts that are crucial for the self-renewal capacity of HSCs [22, 23].

YTHDC1, a member of the YTH domain-containing family of proteins, serves as a nuclear RNA reader for N6-methyladenosine (m^6^A) marks. It exerts distinct regulatory effects on key nuclear RNA processes, including splicing, alternative polyadenylation, nuclear export, and decay, in an m^6^A-dependent manner [24, 25]. These functions are critical for the control of gene expression at the post-transcriptional level [24]. The critical role of N6-methyladenosine in the regulation of HSCs maintenance and function suggests a consequential involvement in leukemia pathogenesis. Accordingly, it is well-documented that m^6^A modifications are significantly associated with leukemia development, particularly in the context of acute myeloid leukemia (AML) [26]. Furthermore, the dysregulation of YTHDC1, an m^6^A reader protein, has been implicated in the pathogenesis of AML and in the maintenance of leukemia stem cells (LSCs) [25, 27].

YTHDC1, alternatively termed YT521-B, is a nuclear-localized protein found within YT bodies, which are regions of active transcription associated with RNA processing speckles [28]. This protein has been recognized for its interaction with the splicing factor SRSF3, thereby playing a role in the modulation of RNA splicing [29]. Additionally, YTHDC1 interacts with NXF1, a principal receptor involved in the nuclear export of mRNAs and non-coding RNAs (ncRNAs) that have been modified by N6-methyladenosine [30]. Recent research has highlighted the critical role of YTHDC1 in the maintenance of heterochromatin integrity and genomic stability in embryonic stem cells during early developmental stages. This is achieved through the YTHDC1-mediated gene silencing of specific retrotransposons, including long interspersed element-1 (LINE-1), intracisternal A particles (IAPs), and endogenous retrovirus-K (ERVK) elements. Notably, these RNA transcripts are subject to m^6^A methylation within mouse embryonic stem cells [31, 32].

Despite its established significance in the realm of RNA biology, the specific functions and mechanisms of YTHDC1 within adult hematopoietic stem cells (HSCs) remain to be elucidated. This study presents a comprehensive examination of YTHDC1, a nuclear protein, and its potential role in the maintenance of adult HSCs.

## Results

### YTHDC1 is required for steady-state HSCs and hematopoiesis

**Fig. 1:**
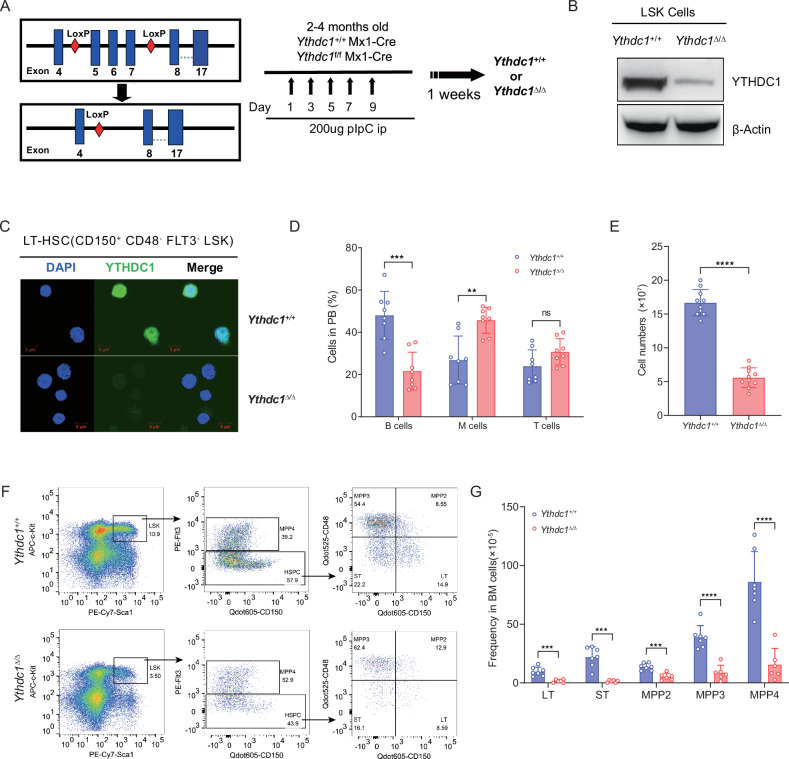
YTHDC1 is required for steady-state HSCs and Hematopoiesis. **A**. The floxed *Ythdc1* experimental schematic for the generation of mice with HSC-specific deletion of *Ythdc1*. *Ythdc1*^flox/flox^ mice were crossed with the interferon-inducible transgenic Mx1-Cre to generate *Ythdc1*^+/+^ Mx1-Cre mice and *Ythdc1*^fl/fl^ Mx1-Cre mice. Animals were then treated with intraperitoneal (i.p.) injections of 300 μg of pIpC every other day for a total of five times to delete the *Ythdc1* alleles. *Ythdc1*^Δ/Δ^ denotes mice with HSC-specific deletion of *Ythdc1*, whereas *Ythdc1*^+/+^ denotes mice without *Ythdc1* deletion as the experimental control. **B** Western blotting analysis of YTHDC1 expression in LSK (Lin^-^Sca1^+^c-Kit^+^) cells from *Ythdc1*^+/+^ and *Ythdc1*^Δ/Δ^ mice; β-Actin was used as a loading control. **C** Immunofluorescence staining of YTHDC1 (green) in the LT-HSCs of *Ythdc1*^+/+^ and *Ythdc1*^Δ/Δ^ mice. Nuclei are stained with DAPI (blue). **D** Composition of myeloid (CD11b^+^), B (B220^+^), and T (CD4^+^/CD8^+^) cells in peripheral blood from *Ythdc1*^+/+^ and *Ythdc1*^Δ/Δ^ mice (*n* = 8 mice per genotype). **E** Whole bone marrow cellularity of *Ythdc1*^+/+^ and *Ythdc1*^Δ/Δ^ mice 1 week after pIpC treatment (*n* = 10 mice per genotype). **F** FACS analysis of LSK, LT-HSCs (CD150 ^+^ CD48^-^ FlK2^-^ LSK), ST-HSCs (CD150^-^CD48^-^ FlK2-LSK), MPP2, MPP3 and MPP4 in *Ythdc1*^+/+^ and *Ythdc1*^Δ/Δ^ BM cells. Representative FACS plots are shown in the panel. **G** Frequency of LT-HSCs, ST-HSCs, MPP2, MPP3 and MPP4 in the bone marrow cells (*Ythdc1*^+/+^, *n* = 7; *Ythdc1*^Δ/Δ^, *n* = 6). Data represent the mean ± SD from n (described above) independent experiments. **P < 0.01; ***P < 0.001; ****P < 0.0001; ns, not significant.

To elucidate the function of YTHDC1 in adult hematopoietic stem cells (HSCs), we employed Mx1-Cre transgenic mouse lines crossed with *Ythdc1*^fl/fl^ mice to achieve specific depletion of YTHDC1 in adult HSCs within the murine bone marrow (Fig. 1A). The efficiency of YTHDC1 knockout in bone marrow HSCs of *Ythdc1*^fl/fl^ Mx1-Cre mice was confirmed through various validation methods (hereafter, *Ythdc1*^fl/fl^ Mx1-Cre mice treated with pIpC are referred to as *Ythdc1*^Δ/Δ^, and *Ythdc1*^+/+^ Mx1-Cre mice treated with pIpC are denoted as *Ythdc1*^+/+^) (Fig. 1B, C). A significant reduction in the counts of mature peripheral blood cells, including white blood cells (WBCs), red blood cells (RBCs), and platelets (PLTs), was observed in *Ythdc1*^Δ/Δ^ mice (Fig. S1C, D, and E). Additionally, an increased proportion of myeloid cells and a decreased proportion of B cells in the peripheral blood were noted in *Ythdc1*^Δ/Δ^ mice (Fig. 1D). Moreover, *Ythdc1*^Δ/Δ^ mice exhibited markedly reduced bone marrow cellularity compared to *Ythdc1*^+/+^ mice (Fig. 1E and Fig. S1B). Flow cytometry analysis revealed that both the proportion and absolute numbers of long-term HSCs (LT-HSCs, also known as SLAM-HSCs), short-term HSCs (ST-HSCs), and multipotent progenitors (MPPs) were significantly diminished in *Ythdc1*^Δ/Δ^ mice relative to *Ythdc1*^+/+^ control mice (Fig. 1F, G; Fig. S1H). Consistent with these findings, *Ythdc1*^Δ/Δ^ mice displayed a substantial decrease in the number and frequency of hematopoietic granulocyte/monocyte progenitors (GMP), common myeloid progenitors (CMP), megakaryocyte/erythroid progenitors (MEP), and common lymphoid progenitors (CLP) compared to control mice (Fig. S1A, F, G, and I, J). Collectively, these data underscore the essential role of YTHDC1 in maintaining hematopoietic balance and HSC homeostasis.

### YTHDC1 is essential for LT-HSC survival and quiescence

**Fig. 2:**
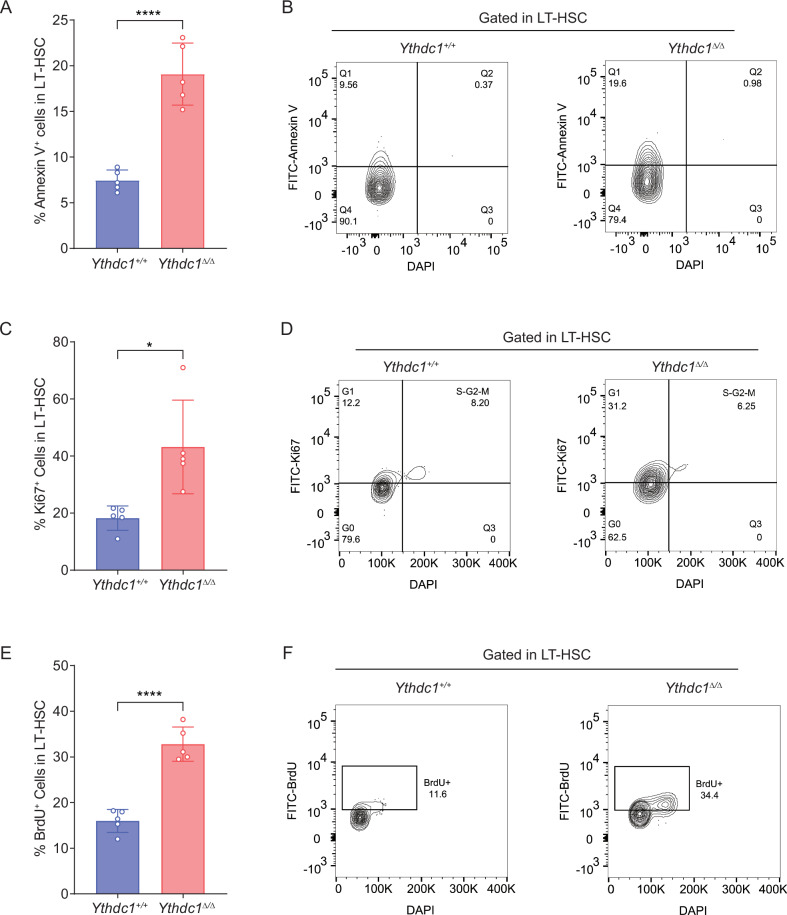
YTHDC1 is essential for LT-HSC survival and quiescence. **A** Frequency of apoptotic cells (ANNEXIN V^+^) in the LT-HSC from *Ythdc1*^+/+^ and *Ythdc1*^Δ/Δ^ mice (*n* = 5 mice per genotype). **B** Representative FACS plots of apoptotic cells (ANNEXIN V^+^) in the LT-HSC from *Ythdc1*^+/+^ and *Ythdc1*^Δ/Δ^ mice(*n* = 5 mice per genotype). **C** Frequency of Ki67^+^ cells in the LT-HSC from *Ythdc1*^+/+^ and *Ythdc1*^Δ/Δ^ mice (*n* = 5 mice per genotype). **D** Representative FACS plots of Ki67^+^ cells in the LT-HSC from *Ythdc1*^+/+^ and *Ythdc1*^Δ/Δ^ mice (*n* = 5 mice per genotype). **E** Frequency of BrdU positive cells in the LT-HSC from *Ythdc1*^+/+^ and *Ythdc1*^Δ/Δ^ mice (*n* = 5 mice per genotype). **F** Representative FACS plots of BrdU positive cells in the LT-HSC from *Ythdc1*^+/+^ and *Ythdc*1^Δ/Δ^ mice (*n* = 5 mice per genotype). Data represent the mean ± SD from *n* (described above) independent experiments. *P < 0.05; ****P < 0.0001.

To gain a deeper understanding of the mechanisms underlying the reduced HSC pool following *Ythdc1* deletion, we performed comprehensive analyses of apoptosis, cell cycle, and cell proliferation in long-term HSCs (LT-HSCs). Our results demonstrated a significant increase in the frequency of apoptotic cells in *Ythdc1*^Δ/Δ^ HSCs compared to *Ythdc1*^+/+^ controls (Fig. 2A, B). Treatment with the apoptosis inhibitor Z-VAD, but not with ferroptosis inhibitors Fer-1, Lip1, DFO, or Nec-1, effectively reduced the frequency of apoptosis in *Ythdc1*^Δ/Δ^ bone marrow cells after a 24-h culture period (Fig. S4E). Ki67 staining revealed a marked increase in the proportion of cycling cells (Ki67^+^) among *Ythdc1*^Δ/Δ^ LT-HSCs, with approximately 42% positivity in *Ythdc1*^Δ/Δ^ cells versus approximately 18% in *Ythdc1*^+/+^ cells (Fig. 2C, D). Additionally, a three-day bromodeoxyuridine (BrdU) labeling assay confirmed a higher frequency of proliferating cells within the *Ythdc1*^Δ/Δ^ LT-HSC population (BrdU^+^ ; approximately 34% in *Ythdc1*^Δ/Δ^vs. approximately 16% in *Ythdc1*^+/+^) (Fig. 2E, F). Collectively, these findings suggest that YTHDC1 is crucial for the preservation of adult HSC survival and the maintenance of their quiescent state.

### YTHDC1 deficiency impairs HSC self-renewal

**Fig. 3:**
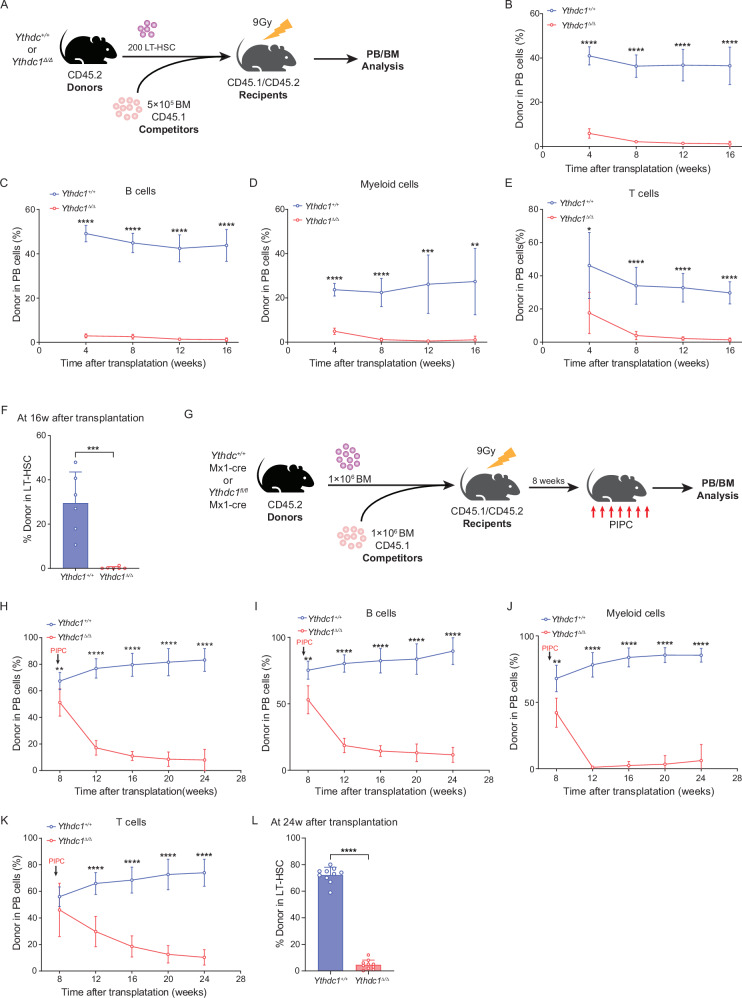
YTHDC1 deficiency impairs HSC self-renewal. **A** Experimental scheme for stem cell competitive transplantation with *Ythdc1*^+/+^ and *Ythdc1*^Δ/Δ^ HSCs (results in Fig. 3B–F). **B** Percentage of donor-derived PB cells at the indicated time points in HSC competitive transplantation assay (*n* = 6 mice per group). **C** Percentage of donor-derived B cells at the indicated time points in HSC competitive transplantation assay (*n* = 6 mice per group). **D** Percentage of donor-derived M cells at the indicated time points in HSC competitive transplantation assay (*n* = 6 mice per group). **E** Percentage of donor-derived T cells at the indicated time points in HSC competitive transplantation assay (*n* = 6 mice per group). **F** Percentage of donor-derived LT-HSCs 16 weeks after transplantation (*n* = 6 mice per group). **G** Experimental scheme for BM competitive transplantation with *Ythdc1*^+/+^ Mx1-Cre and *Ythdc1*^fl/fl^ Mx1-Cre BM cells (results in Fig. 3H–L). Please note: the inducible deletion of Ythdc1 in donor-derived hematopoietic cells was conducted 8 weeks after transplantation. **H** Percentage of donor-derived PB cells at the indicated time points in BM competitive transplantation assay (*n* = 6 mice per group). **I** Percentage of donor-derived B cells at the indicated time points in BM competitive transplantation assay (*n* = 6 mice per group). **J** Percentage of donor-derived M cells at the indicated time points in BM competitive transplantation assay (*n* = 6 mice per group). **K** Percentage of donor-derived T cells at the indicated time points in BM competitive transplantation assay (*n* = 6 mice per group). **L** Percentage of donor-derived LT-HSCs 24 weeks after transplantation (*n* = 6 mice per group). Data represent the mean ± SD from *n* (described above) independent experiments. *P < 0.05; **P < 0.01; ***P < 0.001; ****P < 0.0001.

To elucidate the cell-intrinsic effects of *Ythdc1* deletion on hematopoietic stem cell function, we conducted a competitive repopulation assay. Two hundred long-term HSCs (LT-HSCs), identified by CD45.2 marker, were sorted from donor mice with either *Ythdc1*^+/+^ Mx1-Cre or *Ythdc1*^fl/fl^ Mx1-Cre genotypes. These mice were initially injected with polyinosinic-polycytidylic acid (pIpC) to induce *Ythdc1* deletion in the hematopoietic system. The sorted LT-HSCs were then transplanted into lethally irradiated congenic recipient mice (CD45.1/CD45.2), along with 5 × 10^5^ competitive bone marrow (BM) cells from CD45.1 mice (Fig. 3A). Unlike *Ythdc1*^+/+^ cells, which demonstrated stable long-term multi-lineage reconstitution in the recipient mice, *Ythdc1*^Δ/Δ^ HSC showed a substantial reduction, approximately 40-fold, in their long-term repopulating capacity at 16 weeks post-transplantation (Fig. 3B–F).

To validate these findings, BM cells from *Ythdc1*^+/+^ Mx1-Cre or *Ythdc1*^fl/fl^ Mx1-Cre mice were transplanted into recipients without prior *Ythdc1* deletion. Subsequently, at 8 weeks post-transplantation, the recipient mice were administered pIpC to induce *Ythdc1* deletion, followed by an analysis of peripheral blood (PB) and BM chimerism (Fig. 3G). Post-pIpC treatment, there was a significant decrease in the percentage of donor-derived *Ythdc1*^Δ/Δ^ cells (Fig. 3H). *Ythdc1*^Δ/Δ^ HSCs exhibited a marked reduction in their ability to repopulate all three lineages, including B cells myeloid cells, and T cells (Fig. 3I–K). Furthermore, an in-depth analysis of donor-derived cells within the BM subpopulations of the recipient mice revealed that *Ythdc1*^Δ/Δ^ HSCs generated a significantly lower number of LT-HSCs (Fig. 3L). These results confirm that the depletion of YTHDC1 adversely affects both the multi-lineage repopulation capacity and the self-renewal potential of HSCs in a cell-intrinsic manner.

### YTHDC1 is required to sustain HSCs during ageing

**Fig. 4:**
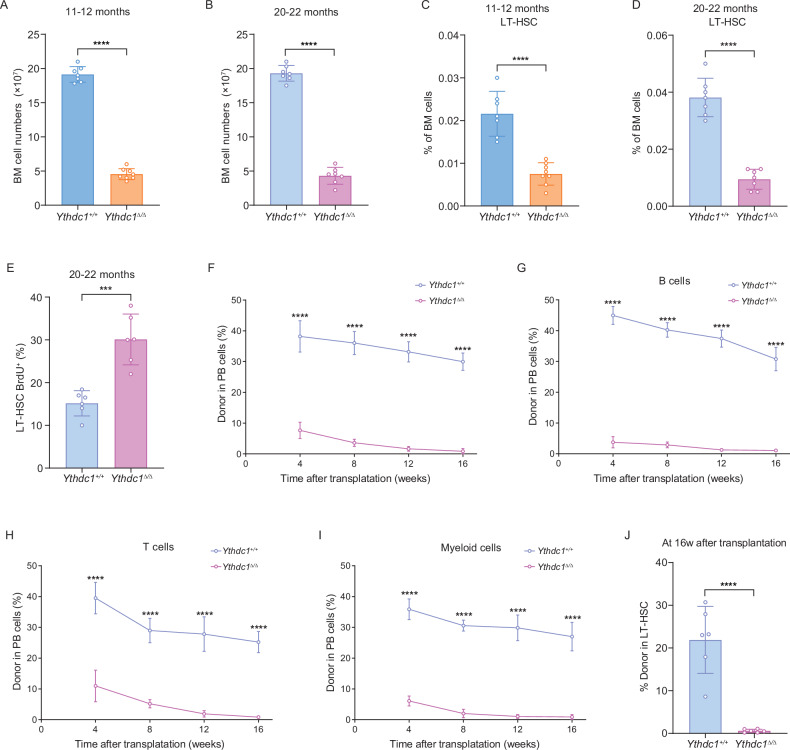
YTHDC1 is required to sustain HSCs during ageing. **A**, **B** The number of bone marrow cells in 11–12-month-old (**A**) or 20–22-month-old (**B**) *Ythdc1*^Δ/Δ^ and *Ythdc1*^+/+^ control mice (*n* = 8 (**A**) or n = 7 (**B**) *Ythdc1*^Δ/Δ^ mice and *n* = 7 (A) or *n* = 7 (**B**) control mice in two independent experiments for each age group). The frequencies of bone marrow HSCs in 11–12-month-old (**C**) and 20–22-month-old (**D**) *Ythdc1*^Δ/Δ^ and *Ythdc1*^+/+^ control mice (*n* = 8 (**C**) or *n* = 7 (**D**) *Ythdc1*^Δ/Δ^ mice and *n* = 7 (C) or *n* = 7 (**D**) control mice in two independent experiments for each age group). **E** The percentage of HSCs from 20–22-month-old mice that incorporated a 72 h pulse of BrdU (*n* = 6 *Ythdc1*^Δ/Δ^ mice and *n* = 6 control mice in two independent experiments). **F** Donor cells from the blood of mice competitively transplanted with bone marrow cells from 20-month-old *Ythdc1*^Δ/Δ^ (*n* = 12 recipients) or control (*n* = 12 recipients) mice (three donors per genotype in three independent experiments). **G** Percentage of donor-derived B cells at the indicated time points in the competitive transplantation assay (*n* = 6 mice per group). **H** Percentage of donor-derived M cells at the indicated time points in the competitive transplantation assay (*n* = 6 mice per group). **I** Percentage of donor-derived T cells at the indicated time points in the competitive transplantation assay (*n* = 6 mice per group). **J**. Percentage of donor-derived LT-HSCs 16 weeks after transplantation (*n* = 6 mice per group). Data represent the mean ± SD from n (described above) independent experiments. ***P < 0.001; ****P < 0.0001.

Our investigation revealed that the expression level of YTHDC1 in hematopoietic stem cells (HSCs) from aged mice (20–22 months old) was significantly elevated compared to that in young mice (2–4 months old) (Fig. S2A, B). This observation prompted us to explore the role of YTHDC1 in the context of aged HSCs. We conducted a comparative study involving mice in two age cohorts: 11–12 months and 20–22 months, to ascertain whether YTHDC1 is crucial for the preservation of HSC function throughout the aging process. Polyinosinic-polycytidylic acid (pIpC) was administered to mice in both age groups to induce YTHDC1 knockout. A significant reduction in bone marrow cellularity was observed in *Ythdc1*^Δ/Δ^ mice relative to control groups, in both the 11–12-month-old (Fig. 4A) and the 20–22-month-old (Fig. 4B) mice. Additionally, the bone marrow of *Ythdc1*^Δ/Δ^ mice contained a significantly diminished number of HSCs at both ages examined (Fig. 4C, D). Notably, in 20–22-month-old *Ythdc1*^Δ/Δ^ mice, the division rate of HSCs, particularly long-term HSCs (LT-HSCs), was considerably higher than in age-matched controls (Fig. 4E). To evaluate the necessity of YTHDC1 for HSC function during aging, we performed competitive transplantation assays into lethally irradiated recipient mice. Bone marrow cells harvested from 20-month-old *Ythdc1*^Δ/Δ^ mice resulted in significantly reduced donor cell reconstitution compared to those from age-matched control mice (Fig. 4F–J). Collectively, these findings suggest that YTHDC1 plays a pivotal role in maintaining the self-renewal capacity of aged HSCs.

### METTL3 mediated RNA m^6^A level loss does not affect survival and function defects of YTHDC1-null HSPCs

**Fig. 5:**
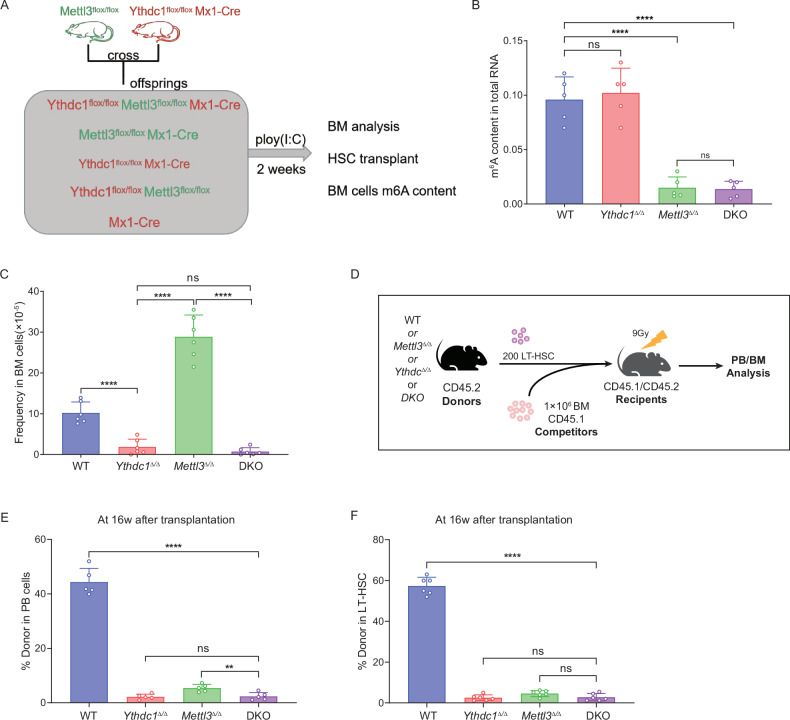
METTL3 mediated RNA m^6^A level loss does not affect survival and function defects of *Ythdc1*-null HSPCs. **A** Experimental scheme for construction of *Mettl3*^Δ/Δ^ *Ythdc1*^Δ/Δ^ mice and stem cell competitive transplantation. **B** Quantification of total RNA m^6^A levels in HSPCs. **C** Frequency of LT-HSCs in the bone marrow cells (*n* = 6 mice per genotype). **D** Experimental scheme for stem cell competitive transplantation (results in Fig. 5E, F). **E** Percentage of donor-derived cells in PB 16 weeks after transplantation (*n* = 6 mice per genotype). **F** Percentage of donor-derived LT-HSCs 16 weeks after transplantation (*n* = 6 mice per group). Data represent the mean ± SD from n (described above) independent experiments. **P < 0.01; ****P < 0.0001; ns not significant.

Previous research has demonstrated that the deletion of the m^6^A methyltransferase *Mettl3* in the adult hematopoietic system results in diminished global m^6^A levels and a three fold increase in hematopoietic stem cell abundance within the bone marrow [19]. We postulate that the reduced frequency of *Ythdc1*-null HSCs, which contrasts with the phenotype observed in *Mettl3*-deficient mice, may not be associated with the m^6^A RNA levels in bone marrow cells. To investigate this hypothesis, we generated double-knockout (DKO) mice by crossing Mx1-cre mice with *Mettl3*^fl/+^ and *Ythdc1*^fl/+^ mice, thereby producing *Mettl3*^fl/fl^ *Ythdc1*^fl/fl^ Mx1-cre mice (Fig. 5A). The m^6^A content in lineage-depleted hematopoietic cells from these double-KO mice, as well as from *Mettl3*^fl/fl^ Mx1-cre mice (*Mettl3*^Δ/Δ^), was significantly reduced compared to control mice or *Ythdc1*^fl/fl^ Mx1-cre mice (*Ythdc1*^Δ/Δ^) (Fig. 5B). The frequency and absolute number of long-term HSCs (LT-HSCs), short-term HSCs (ST-HSCs), and multipotent progenitors (MPP2, MPP3, and MPP4) in the bone marrow of double-KO mice were markedly lower than those in control or *Mettl3*^Δ/Δ^ mice, yet they were similar to the levels observed in *Ythdc1*^Δ/Δ^ mice (Fig. 5C, Fig. S3B, C). Furthermore, the bone marrow cellularity of DKO mice was significantly lower than that of wild-type and *Mettl3*^Δ/Δ^ mice, but it did not differ from that of *Ythdc1*^Δ/Δ^ mice (Fig. S3A).

Subsequently, we conducted a competitive reconstitution assay to evaluate the impact of the combined deletion of *Mettl3* and *Ythdc1* (double-KO) on hematopoietic stem cell self-renewal capabilities in recipient mice (Fig. 5D). Sixteen weeks post bone marrow transplantation, a pronounced decrease in the presence of donor-derived HSCs within the bone marrow and donor-derived cells in the peripheral blood of recipient mice was observed when they were engrafted with HSCs from double-KO mice. This reduction indicates that HSCs from double-KO mice exhibit significantly diminished long-term reconstituting activity compared to those from control mice (Fig. 5E, F). These findings suggest that the role of YTHDC1 in HSC survival and regulation is not influenced by the absence of the m^6^A methyltransferase METTL3 in HSCs.

### YTHDC1 interacts with HP1BP3-mediated microRNA processing in HSPCs

**Fig. 6:**
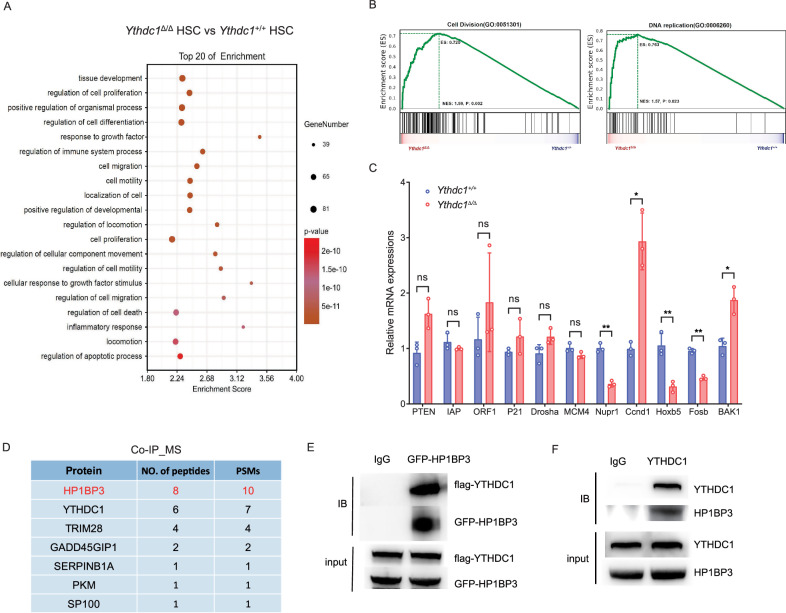
YTHDC1 interacts with Hp1-binding-protein 3 mediated microRNA processing in HSPCs. **A** Gene ontology (GO) enrichment analysis of the transcriptional signature of HSCs in *Ythdc1*^Δ/Δ^ relative to *Ythdc1*^+/+^ group. **B** GSEA analysis of the transcriptional signature of HSCs in *Ythdc1*^Δ/Δ^ relative to *Ythdc1*^+/+^ group. **C** qPCR validation of published YTHDC1 regulated genes expression in *Ythdc1*^Δ/Δ^ LT-HSC and *Ythdc1*^+/+^ LT-HSC (*n* = 3 mice per genotype). **D** Co-IP MS analysis of YTHDC1 interacted proteins in wild type HSPC. **E** HEK293 T cells were co-transfected with plasmids encoding GFP-tagged HP1BP3 and FLAG-tagged YTHDC1. Cell lysates were immunoprecipitated with anti-FLAG M2 agarose and immune complexes were analyzed by immunoblotting using antibodies against FLAG or GFP. **F** HP1BP3/YTHDC1 interaction as revealed by IP assay in FACS sorted wild-type HSPCs (Lin^-^ c-Kit^+^). IgG antibody was used as the negative control. Data represent the mean ± SD from n (described above) independent experiments. *P < 0.05; **P < 0.01; ns, not significant.

To elucidate the molecular mechanisms underlying YTHDC1-mediated hematopoietic stem cell (HSC) homeostasis and regeneration, we conducted RNA sequencing (RNA-seq) analysis on freshly isolated *Ythdc1*^+/+^ and *Ythdc1*^Δ/Δ^ HSCs. Gene ontology (GO) enrichment analysis of 1345 differentially expressed genes (DEGs) revealed significant enrichment of YTHDC1-dependent genes associated with cellular proliferation, cell death regulation, and cell migration in HSCs (Fig. 6A, Fig. S4A). Gene set enrichment analysis (GSEA) further indicated a pronounced upregulation of genes involved in cell division and DNA replication (Fig. 6B). Additionally, the expression of key HSC stemness genes was significantly diminished in YTHDC1-deficient HSCs (Fig. S4B). GSEA also highlighted a significant positive enrichment of the HSC fingerprint gene set in the wild-type HSC transcriptome, whereas the canonical WNT signaling pathway, known for its critical role in HSC maintenance, was not significantly enriched (Fig. S4C, D). Quantitative PCR (qPCR) analysis of a selection of published YTHDC1 downstream target genes, including *Pten*, *IAP*, *ORF1*, *MCM4*, *p21* and *Drosha*, revealed no changes in *Ythdc1*^Δ/Δ^ HSCs compared to *Ythdc1*^+/+^ HSCs (Fig. 6C). In contrast, the cell cycle-related gene *Ccnd1* and the proapoptotic gene *Bak1* were significantly upregulated, while the expression of *Hoxb5*, *Nupr1*, and *FosB*, which are associated with HSC function, was significantly downregulated in *Ythdc1*^Δ/Δ^ HSCs (Fig. 6C). To identify candidate molecules involved in YTHDC1-mediated HSC maintenance, we performed co-immunoprecipitation followed by mass spectrometry (Co-IP-MS) experiments. By comparing these samples with control samples processed using the same methodology, we identified HP1BP3 as a candidate protein specifically associated with YTHDC1 (Fig. 6D). To substantiate this association, we conducted transient transfection and Co-IP experiments, confirming that HP1BP3 interacts with YTHDC1 in a mammalian overexpression system (Fig. 6E, F).

### Loss of YTHDC1 decreases the expression level of some microRNAs which mediated HSCs survival and function

**Fig. 7:**
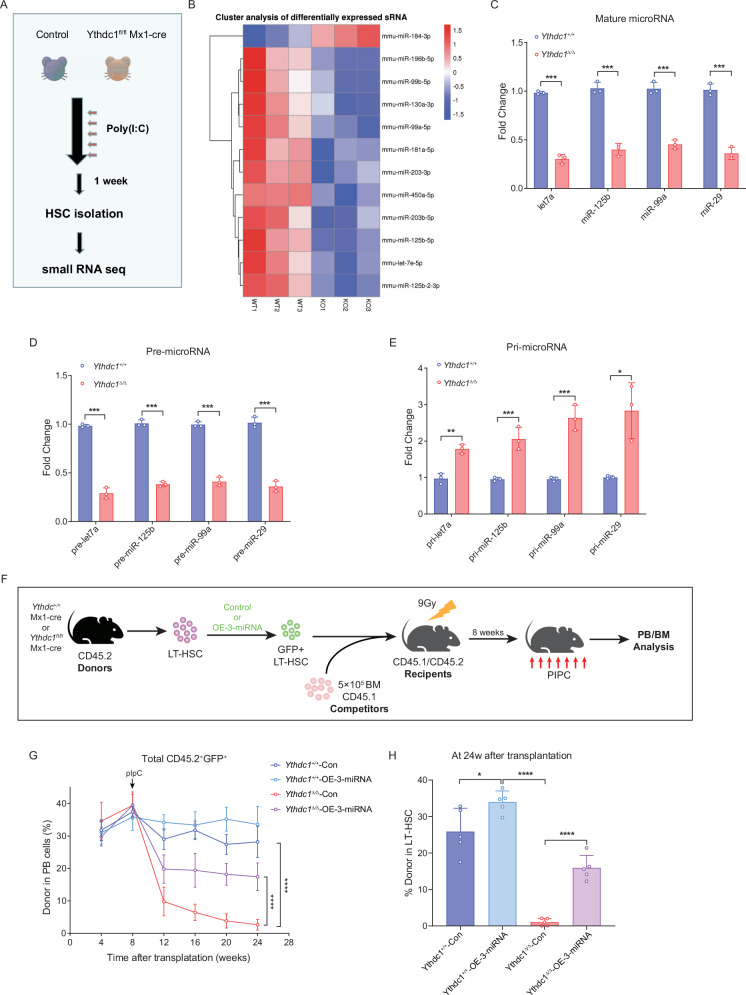
Loss of YTHDC1 decreases the expression level of some microRNAs which mediated HSC survival and function. **A**. Experimental scheme for microRNA seq analysis. **B** Representative heat map of the microRNA expression levels by small RNA seq. The relative expression levels of listed genes in *Ythdc1*^Δ/Δ^ HSCs vs. control HSCs are shown. **C** Quantitative analysis of the expression fold change of mature miRNA by Taqman QPCR between the control and YTHDC1-depleted HSCs (*n* = 3). **D** Quantitative analysis of the expression fold change of pre-miRNA by Taqman QPCR between the control and YTHDC1-depleted HSCs (*n* = 3). **E** Quantitative analysis of the expression fold change of pri-miRNA by Taqman QPCR between the control and YTHDC1-depleted HSCs (*n* = 3). **F** Experimental scheme for ectopic 3-miR HSC competitive transplantation assay. **G** Percentage of donor-derived PB cells at the indicated time points in ectopic 3-miR HSC competitive transplantation assay (*n* = 6 mice per group). Please note: the inducible deletion of *Ythdc1* in donor-derived hematopoietic cells was conducted 8 weeks after transplantation. **H** Percentage of donor-derived LT-HSCs 24 weeks after transplantation (*n* = 6 mice per group). Data represent the mean ± SD from *n* (described above) independent experiments. *P < 0.05; **P < 0.01; ***P < 0.001; ****P < 0.0001.

A previous study has demonstrated that HP1BP3 possesses specific binding activity for primary microRNAs (pri-miRNAs) and facilitates co-transcriptional miRNA processing [33]. In light of this, we conducted small RNA sequencing analysis on freshly isolated *Ythdc1*^+/+^ and *Ythdc1*^Δ/Δ^ HSCs (Fig. 7A). The heatmap analysis revealed significant downregulation of *miR-125b*, *miR-99b*, and *let-7e* in *Ythdc1*^Δ/Δ^ HSCs (Fig. 7B). Additionally, our data indicated that YTHDC1 depletion led to a significant increase in the levels of four endogenous pri-miRNAs and a concomitant reduction in four endogenous mature miRNAs and pre-miRNAs in HSCs (Fig. 7C–E). Prior research has established that *miR-99* knockdown results in an increased percentage of cycling hematopoietic stem and progenitor cells (HSPCs), and *miR-125b* promotes cell survival [34, 35]. We also observed a diminished interaction between HP1BP3 and DROSHA in *Ythdc1*^Δ/Δ^ HSPCs compared to *Ythdc1*^+/+^ HSPCs (Fig. S5B).

To ascertain whether the reduced levels of mature miRNAs in *Ythdc1*^Δ/Δ^ HSCs contribute to aberrant HSC survival and functional deficiencies, we utilized a lentiviral overexpression system to introduce a cluster of *mir-125b*, *mir-99b* and *let-7e* into *Ythdc1*^+/+^ and *Ythdc1*^Δ/Δ^ HSCs and subsequently assessed HSC numbers and functionality (Fig. 7F, Fig. S5A). Competitive transplantation assays were then performed, and the results showed that co-overexpression of *mir-125b*, *mir-99b*, and *let-7e* in *Ythdc1*^Δ/Δ^ HSCs partially restored the reconstitution capacity of donor-derived HSCs in the peripheral blood (Fig. 7G) and the long-term HSC compartment of the recipients compared to control treatments (Fig. 7H). This suggests that the co-overexpressed miRNAs also rescued the functional deficits of YTHDC1-deficient HSCs. Collectively, these findings indicate that mature *mir-125b*, *mir-99b* and *let-7e* serve as functional downstream targets of YTHDC1 in HSCs.

## Discussions

In this study, we delineate a pivotal role for YTHDC1 in the maintenance of adult hematopoietic stem cells. Specifically, we demonstrate that YTHDC1 interacts with HP1BP3, thereby participating in microRNA processing. Our findings reveal that YTHDC1 is cell-autonomously essential for maintaining the survival, quiescence, and self-renewal capacity of HSCs throughout the adult lifespan.

Previous research has established that the depletion of *Mettl3*, an enzyme responsible for the m^6^A methylation of RNA, leads to an increase in hematopoietic stem cells numbers in murine models [19]. Our own prior work has corroborated this by showing that the absence of the m^6^A reader gene *Ythdf2* also results in HSCs expansion [25]. Furthermore, Zhang et al. have reported that the loss of *Ythdf3*, but not *Ythdf1*, induces a mild expansion of the HSCs pool [36]. In contrast, our current data indicate that the loss of *Ythdc1* results in HSCs exhaustion and bone marrow failure in mice, a finding that is congruent with the results presented by Sheng et al. [25] (Figs. 1D–G, Fig. 3A–L, and Fig. S1B–H). Both our study and that of Sheng et al. have discovered that the deletion of YTHDC1 enhances apoptosis in hematopoietic stem and progenitor cells (Fig. 2A, B, Fig. S4E). Additionally, we have demonstrated that the knockout of *Ythdc1* accelerates cell cycle entry in HSCs and elevates the proportion of BrdU-labeled HSCs (Fig. 2C–F). Conversely, the loss of *Ythdc1* inhibits DNA synthesis in leukemia stem cells (LSCs) [25]. The divergent effects of YTHDC1 depletion on DNA synthesis in HSCs versus LSCs suggest that distinct molecular mechanisms may be at play.

YTHDC1, a nuclear-localized reader of N6-methyladenosine (m^6^A)-modified RNA, has been implicated in various aspects of gene expression, including transcription, RNA processing—such as mRNA splicing, polyadenylation, and microRNA maturation—as well as RNA degradation and nuclear export [37–40]. Additionally, YTHDC1 has been shown to interact with the demethylase KDM3B, leading to the demethylation of H3K9me2 at m^6^A RNA-associated chromatin regions [41]. In this study, we identified a novel interaction between YTHDC1 and HP1BP3 in the regulation of microRNA processing (Fig. 6D–F, Fig. 7A–G). Conversely, we also observed that YTHDC1 modulates the expression of MCM2, MCM4, and MCM5 in acute myeloid leukemia (AML) cells by controlling their mRNA stability [25]. However, the precise mechanism underlying the interaction between YTHDC1 and HP1BP3 in microRNA processing remains to be elucidated. We hypothesize that the absence of YTHDC1 may disrupt microRNA maturation in YTHDC1-null HSCs. It remains uncertain whether YTHDC1-mediated microRNA maturation is directly through the recognition of m^6^A sites on primary microRNA transcripts. Furthermore, the transcription and stability of microRNAs, as well as the epigenetic regulation of microRNA promoters in YTHDC1 knockout HSCs, were not examined in this study and warrant further investigation.

Previous research has documented that specific microRNAs, including *miR-99b*, *let-7e*, *miR-125a*, and *miR-125b*, are enriched in long-term HSCs [34, 35, 42, 43]. Ooi et al. have demonstrated that the overexpression of *miR-125b* in HSCs augments their functionality and selectively enriches for lymphoid-balanced and lymphoid-biased HSC subsets [35]. Furthermore, they have identified *Bmf* and *Klf13* as downstream targets of *miR-125b*, which are implicated in the regulation of apoptosis. Our findings corroborate these observations, as we have confirmed the upregulation of the proapoptotic gene *Bak1* in YTHDC1-deficient HSCs relative to control cells, suggesting that elevated apoptosis in these cells may be a contributing factor to the observed alterations in hematopoiesis (Fig. 6C). Our data indicate that the overexpression of the miR-cluster *99b/let-7e/125b*, facilitated by a lentiviral chimeric miRNA cluster platform (OE-3-miR), can partially ameliorate the survival and functional deficiencies observed in *Ythdc1*^Δ/Δ^ HSCs [44, 45].

**Fig. 8:**
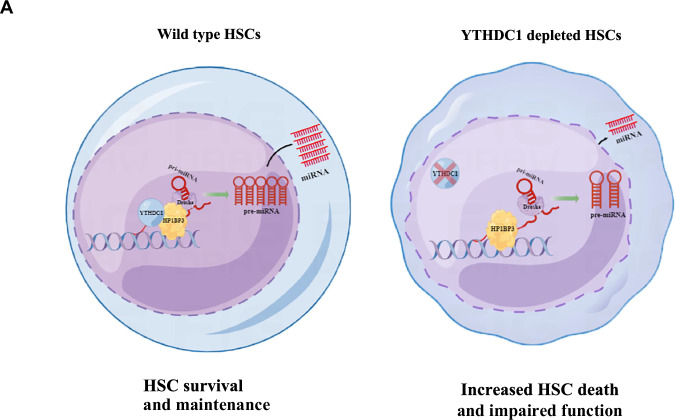
Schematic depicting YTHDC1-mediated microRNA processing in HSCs is essential for HSCs survival and maintenance. **A** YTHDC1 is essential for HSC survival and maintenance (left), delpetion of YTHDC1 in HSC impairs HSC function and promotes apoptosis.

In conclusion, our research has uncovered a novel role for YTHDC1 in the regulation of HSCs quiescence, survival, and self-renewal. Our findings suggest that YTHDC1, in part, exerts these effects through the modulation of microRNA processing, facilitated by its interaction with HP1BP3 within the HSC compartment (Fig. 8A). Nonetheless, further investigation is warranted to fully elucidate the intricate roles of YTHDC1 in the transcription, maturation, and stability of microRNAs, and to determine whether these regulatory mechanisms are dependent or independent of its m^6^A recognition capabilities.

## Materials and methods

### Mice

Targeted embryonic stem (ES) cell clones for the generation of *Ythdc1* and *Mettl3* knockout (KO) first chimeric mice were sourced from the European Conditional Mouse Mutagenesis Program (EUCOMM). The progeny of chimeric males were subsequently bred with Flpe deleter mice to excise the neomycin (Neo) cassette.*Ythdc1*^fl/fl^ mice were then mated with Mx1-Cre transgenic mice to produce *Ythdc1*^fl/+^ Mx1-Cre and *Ythdc1*^fl/fl^ Mx1-Cre offspring. *Ythdc1*^fl/fl^ mice were derived from crosses between *Ythdc1*^fl/fl^ chimeric mice and flipper mice, which facilitated the removal of the FRT-flanked selection cassette. All mouse strains were maintained on a C57BL/6 genetic background. For the competitive transplantation assays, recipient mice were either B6.SJL-PtprcaPep3b/Boy (CD45.1) mice or CD45.1/CD45.2 heterozygotes, which were the first generation offspring from a cross between C57BL/6 and B6.SJL-PtprcaPep3b/Boy mice. The Animal Care and Ethics Committee at Hangzhou Normal University provided approval for all animal experiments conducted in this study. The mice were housed in a specific pathogen-free environment and provided with a standard diet.

### Flow Cytometry

Prepared samples were analyzed on an LSRFortessa^TM^ cell analyzer (BD Biosciences) or sorted on an MoFlo Astrios EQ cell sorter (Beckman coulter). Detailed methods and antibodies were as our lab paper described previously. The antibody cocktail including biotinylated CD11b (M1/70, Biolegend), Gr1 (RB6-8C5, Biolegend), CD4 (RM4-5, Biolegend), CD8 (53-6.7, Biolegend), Ter119 (Ter-119, Biolegend), and B220 (RA3-6B2, Biolegend) was used to define lineage cells. APC/Cyanine 7 Streptavidin (BioLegend), c-Kit (2B8, eBioscience), Sca1 (D7, Biolegend), CD48 (HM48-1, BioLegend), CD150 (TC15-12F12.2, BioLegend), CD34 (RAM34, eBioscience), Flt3 (A2F10, eBioscience), CD16/32 (93, eBioscience), IL-7R (A7R34, eBioscience), CD45.2 (104, BioLegend), CD45.1 (A20, BioLegend), CD4 (RM4-5, BioLegend), CD8 (53-6.7), B220 (RA3-6B2, BioLegend), and CD11b (M1/70, BioLegend) were used where indicated.

### Isolation of murine HSC

Donor mice at 8 weeks of age were euthanized by cervical dislocation and bone marrow (BM) cells were isolated by crushing the bones from hind legs, forelegs, pelvis, spine and sternum. BM cells were firstly enriched for HSCs by anti-CD117 conjugated magnetic beads (Miltenyi) and followed by FACS sorting.

### Competitive transplantation assay

Sorted 200 LT-HSC cells from donor mice (CD45.2) were mixed with 5×10^5^ BM MNCs from age-matched WT mice (competitor, CD45.1) and injected into lethally irradiated (8 Gy) recipient mice (CD45.1/CD45.2).

### Immunofluorescence staining

LT-HSCs were sorted to polylysine-coated glass coverslips, then fixed with 4% paraformaldehyde (PFA), permeabilized with 0.4% Triton X-100 detergent, and blocked with 3% BSA. Coverslips were incubated with antibodies against YTHDC1 (1:100; Proteintech, 14392-1-AP) overnight at 4 °C. The samples were then washed with PBS for three times, followed by incubation with secondary antibodies (1:400; Thermo Scientific, A21206) for 1 h at RT. Nuclei were stained with DAPI.

### Hematopoietic cells culture

Murine bone marrow (BM) cells or sorted HSPC were cultured with serum-free medium (09650, STEM CELL) supplemented with SCF (50 ng/mL), TPO (50 ng/mL), and 1% penicillin-streptomycin in 96-round-well plates. In vitro apoptosis assay of BM cells, cells were treated with apoptosis inhibitor or ferroptosis inhibitor as indicated. HSPC cultured for 12 h were then infected with lentivirus. The transduced cells were transplanted into lethally irradiated recipient mice after 2 days of culture.

### Lentivirus production

Lentivirus was produced in 293 T cells after transfection of 7 mg shRNA plasmid, 5 mg pspAX2 helper plasmid, and 3 mg pMD2G by using polyethylenimine. Cells in DMEM medium with 15% Fetal Bovine Serum (Excell Bio) were replaced with fresh medium 12 h after transfection. Collected the virus supernatant 48 h after transfection and concentrated by centrifugation of the medium at 25,000 rpm for 2.5 h at 4 °C. The virus pellet was dissolved in sterile PBS. Lentiviral titers were assessed and calculated by the transfection of c-kit positive cells. 2 × 10^5^ sorted LSK cells were transduced with the lentivirus.

### Lentivirus transduction

Freshly sorted LSK cells were resuspended in hematopoietic cells culture medium as described above. Virus particles were added to cell suspension according to the determined titer (~ 90% transduction efficiency). Then the mixture of cells and virus was spinoculated for 2 h at 600 g at room temperature. After 2 days culture, transduced GFP positive HSPC cells were sorted for further transplantation.

### HSC cell cycle and apoptosis analysis

Ki67 staining was used to determine cell cycle status. BrdU incorporation assay was utilized to determine the proliferation rate of HSCs. For in vivo analysis, BrdU (100 mg/kg; BD Biosciences) was injected i.p. into *Ythdc1*^+/+^ and *Ythdc1*^Δ/Δ^ mice for 3 days. BrdU incorporation was determined by FACS analysis using the BrdU Flow Kit (BD Biosciences) according to the manufacturer’s instructions.

### RNA purification and Real-Time PCR

Total RNA was extracted from freshly isolated cells using a RNeasy kit (Qiagen). Complementary DNA was then synthesized from the RNA using a HiScript II One Step RT-PCR Kit (Vazyme Biotech Co.Ltd) for First-Strand cDNA Synthesis according to the manufacturer’s instructions. The quantitative RT-PCR analysis was conducted using a CFX96 Real-Time System (Bio-Rad). The relative expression levels of the genes of interest were calculated using the relative delta-delta-Ct method. The expression of β-actin was used as the internal control.

### TaqMan qPCR

A miRNA 1st Strand cDNA Synthesis Kit (Vazyme) was used to synthesize miRNA cDNA. The TaqMan qPCR for miRNA quantification was carried out following the manufacturers’ protocols. The total reaction volume was 10 μL, which included 1×TaqMan Fast qPCR Master Mix, 0.2 μM TaqMan probe, 0.8 μM primers, and synthesized miRNA cDNA. Each reaction was performed in triplicate and incubated at 96 °C for 10 min, followed by 50 cycles of 95 °C for 10 s and 60 °C for 30 s.

### Co-IP/MS

Purified HSPC were lysed for half an hour in IP lysis buffer containing protease inhibitor. Subsequently, 3 μg antibodies were added to the protein samples, which were 500 μg in total. This mixture was gently rotated and incubated for 2 h at 4 °C. Then, the proteins were further incubated with 30 μl of protein A/G agarose overnight at 4 °C, the beads were washed four times with IP lysis buffer. After that, the samples were subjected to LC-MS/MS analysis, which was conducted and evaluated by PTM BIO company in Hangzhou.

### RNA-sequencing (RNA-seq) and data analysis

cDNA was ligated with sequencing adapters according to standard illumine Hiseq 2000/2500 protocols. Sequencing libraries were generated using NEBNext® Ultra™ RNA Library Prep Kit for Illumina® (NEB, USA) following manufacturer’s recommendations and index codes were added to attribute sequences to each sample. The clustering of the index-coded samples was performed on a cBot Cluster Generation System using TruSeq PE Cluster Kit v3-cBot-HS (Illumia) according to the manufacturer’s instructions. After cluster generation, the library preparations were sequenced on an Illumina Hiseq platform and 125 bp/150 bp paired-end reads were generated. Raw data (raw reads) of fastq format were first processed through in-house perl scripts. In this step, clean data (clean reads) were obtained by removing reads containing adapter, reads containing ploy-N and low-quality reads from raw data. At the same time, Q20, Q30 and GC content of the clean data were calculated. All the downstream analyses were based on clean data with high quality. The sequencing reads were mapped to the mouse genome (mm10) using Hisat2 (version:2.0.4). Stringtie (version:1.3.0) was used to calculate fragments per kilobase of exon model per million mapped reads (FPKM) values. Omigen (Hangzhou) Inc provide assistance for the data processing. EdgeR was used to analyze differential genes based on two-fold changes and *p* ≤ 0.05. HSC stemness genes were summarized in a previous study [46].

### Gene set enrichment analysis (GSEA)

Gene set enrichment analysis (GSEA) analyses were performed using the tool available at http://www.broadinstitute.org/gsea/index.jsp. In brief, fold change (log2) in gene expression from two experimental conditions were calculated and the list was then used as a ranked list in the Pre-Ranked function of the GSEA software. The statistical significance of GSEA was set as FDR < 0.1, which is more stringent than the default setting of GSEA software. The HSC fingerprint gene set was described in previous study [47, 48].

### MicroRNA-sequencing

Small RNA was extracted from HSC using the miRNeasy Mini Kit (Qiagen). Library construction, quality Control, and sequencing were conducted by Novogene company in Beijing. Briefly, 3’ and 5’ adapters were ligated to 3’ and 5’ end of small RNA, respectively. Then the first strand cDNA was synthesized after hybridization with reverse transcription primer. The double-stranded cDNA library was generated through PCR enrichment. After purification and size selection, libraries with insertions between 18~40 bp were ready for sequencing on Illumina sequencing with SE50. The library was checked with Qubit and real-time PCR for quantification and bioanalyzer for size distribution detection. Quantified libraries will be pooled and sequenced on Illumina platforms.

### Statistical analyses

The exact sample size (n) for each experimental group/condition was given in figure legends. Unpaired two-tailed Student’s *t*-test was utilized to determine statistical significance; *P* values less than 0.05 were considered significant. No statistical methods were used to predetermine the sample size. Mice were randomly allocated to experimental groups. No blinding method was used for injection. There was no animal exclusion criteria. The variance was similar between the groups that were being statistically compared. Statistics were performed using Prism 8 (GraphPad, San Diego, USA).

## Supplementary information

Supplementary figure and figure legend

WB origin gel

## Data Availability

RNA-seq data have been deposited in NCBI Gene Expression Omnibus (GEO) database under accession number GSE208626 (private status: avqpimkerdynfeh).
