# Supplementary material for: YTHDC1-mediated microRNA maturation is essential for hematopoietic stem cells maintenance
Source: Cell Death Discov. 2024 Oct 16;10:439. doi: 10.1038/s41420-024-02203-z (PMC11484846; doi:10.1038/s41420-024-02203-z)
Supplement: Supplementary file 1 — Supplementary figure and figure legend [file 41420_2024_2203_MOESM1_ESM.pdf]

Figure S1 Conditional loss of YTHDC1 in hematopoietic cells leads to hematopoiesis and bone marrow failure

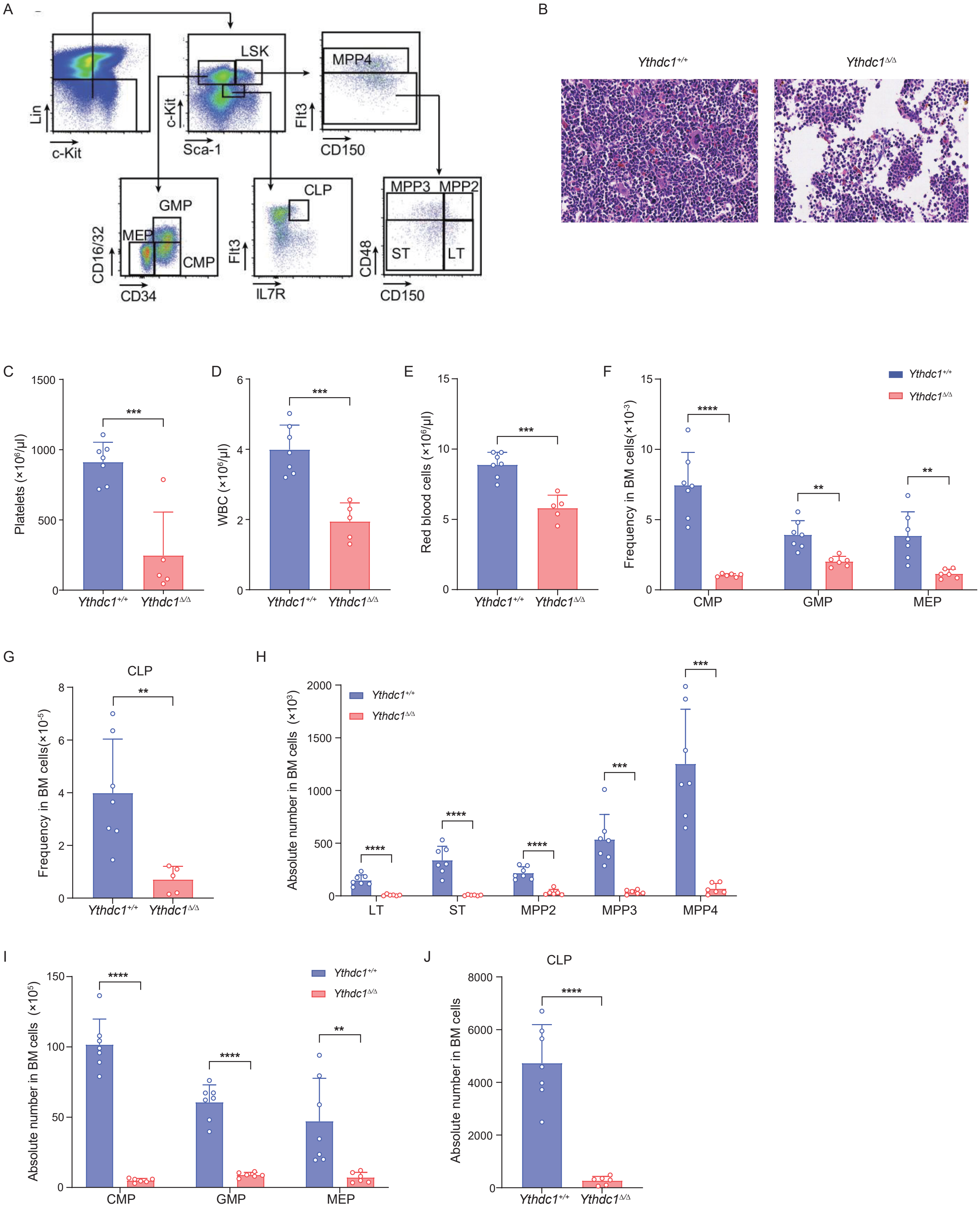

Figure S2 YTHDC1 is required to sustain HSCs during ageing

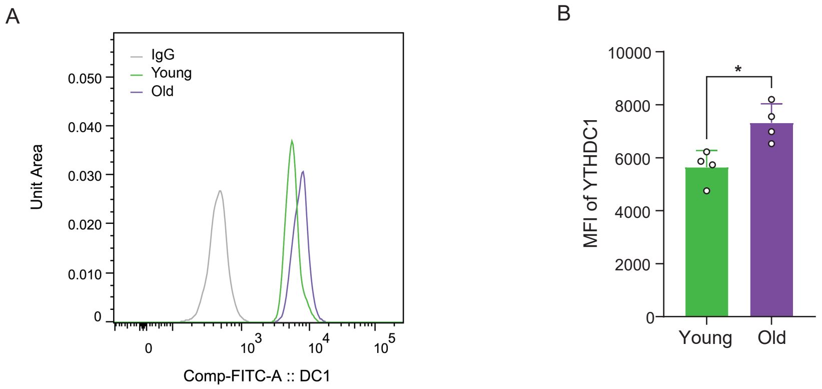

Figure S3 YTHDC1 and METTL3 double knock-out mice displays dissimilar phenotype with METTL3 knockout mice

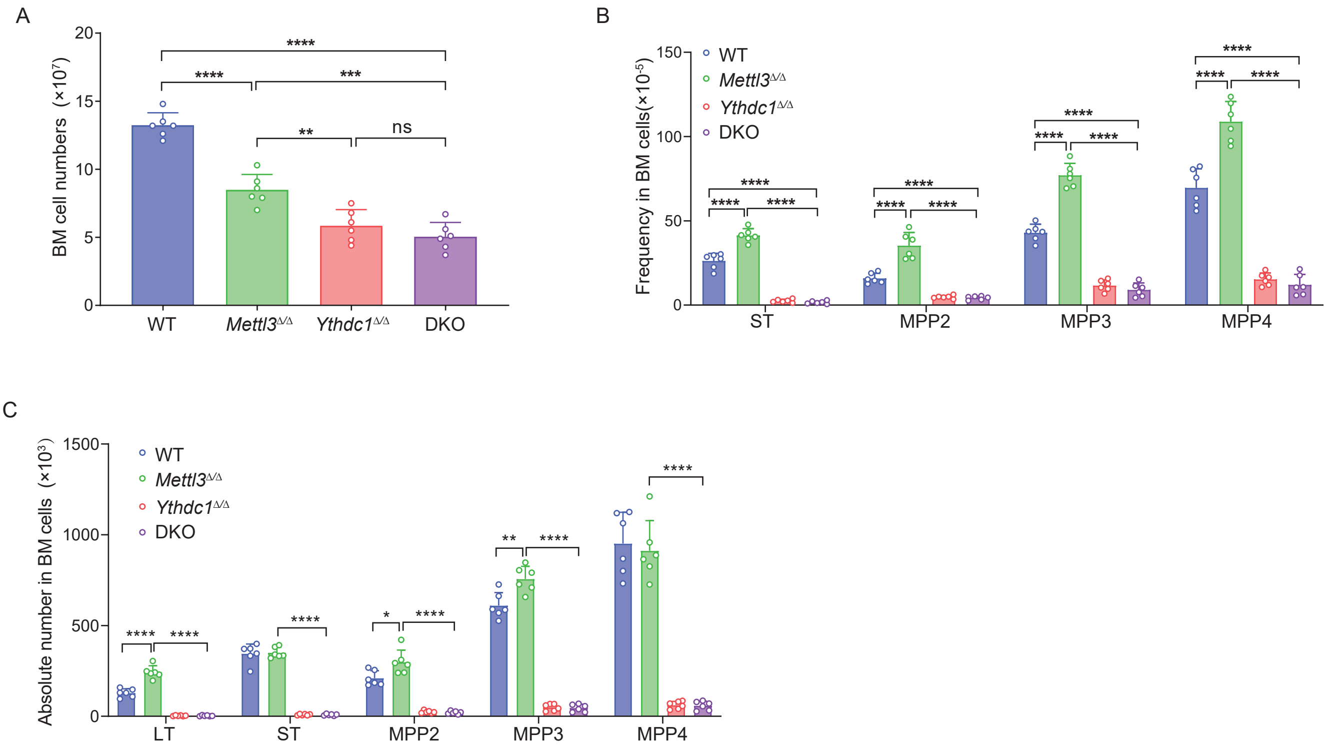

Figure S4 Deletion of YTHDC1 impairs HSC stemness and affects HSC apoptosis

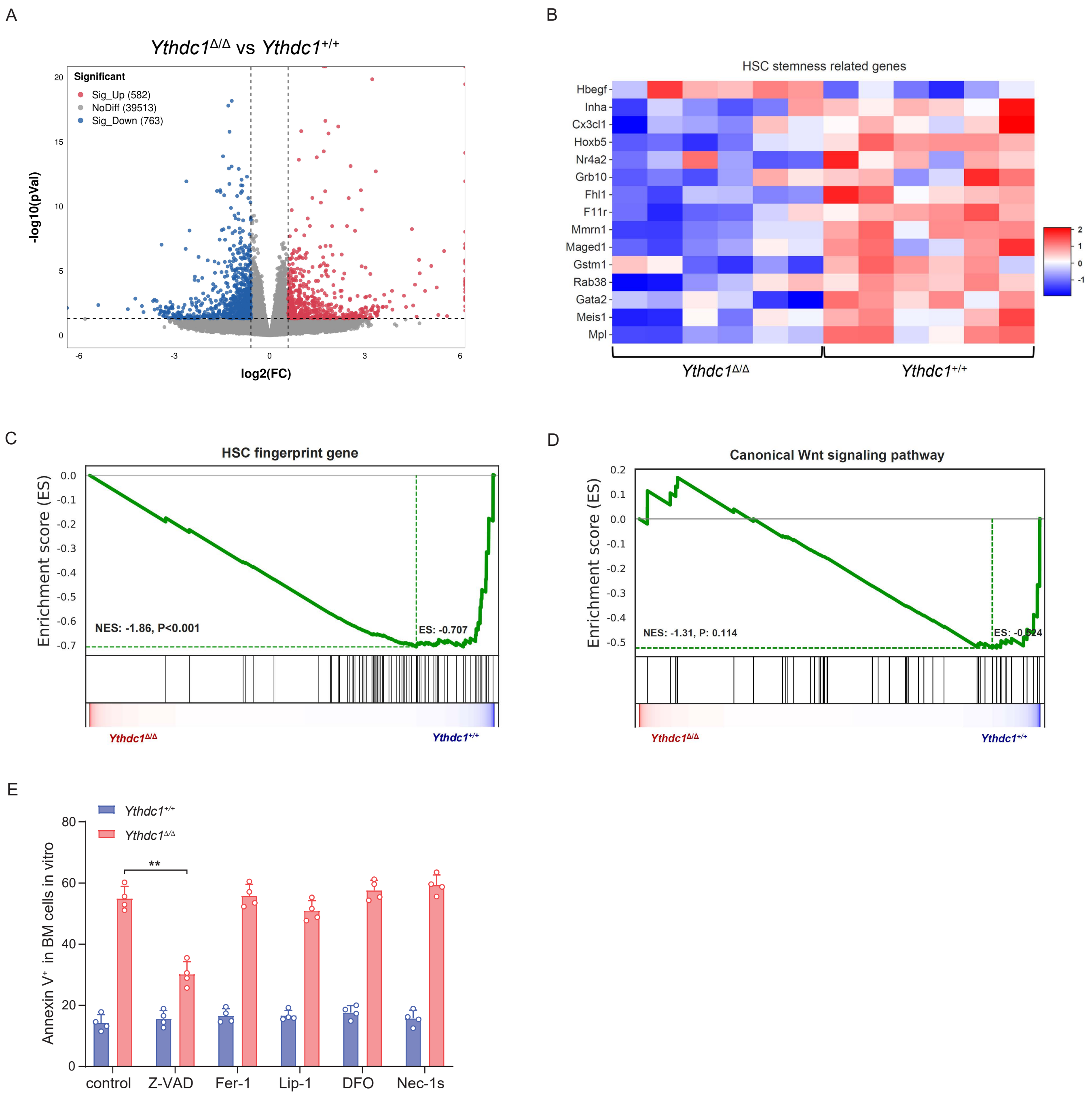

Figure S5 Deletion of YTHDC1 impairs HSC stemness and affects HSC apoptosis

A

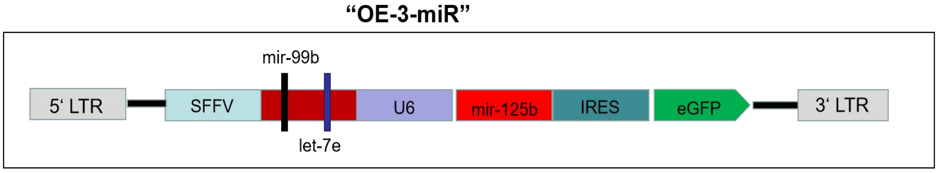

B

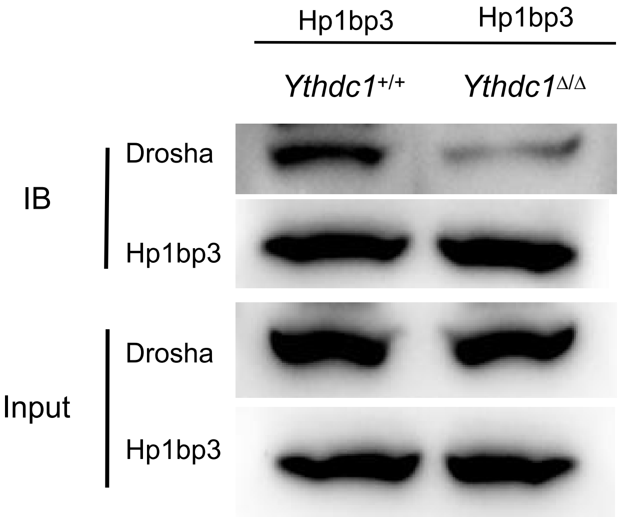

Figure S1. Conditional loss of YTHDC1 in hematopoietic cells leads to hematopoiesis and bone marrow failure.

A. Gating strategy of HSCs analysis and sorting.

B. Histological analysis of hematoxylin and eosin-stained sternum from *Ythdc1*$^{+/+}$ and *Ythdc1*$^{\Delta/\Delta}$ mice, bar = 50μM.

C. Absolute number of platelets (PLT) in peripheral blood from *Ythdc1*$^{+/+}$ (n=7) and *Ythdc1*$^{\Delta/\Delta}$(n=5).

D. Absolute number of white blood cells (WBC) in peripheral blood from *Ythdc1*$^{+/+}$ (n=7) and *Ythdc1*$^{\Delta/\Delta}$(n=5).

E. Absolute number of red blood cells (RBC) in peripheral blood from *Ythdc1*$^{+/+}$ (n=7) and *Ythdc1*$^{\Delta/\Delta}$(n=5).

F. Frequency of CMP, MEP and GMP in the bone marrow cells (*Ythdc1*$^{+/+}$, n=7; *Ythdc1*$^{\Delta/\Delta}$, n = 6).

G. Frequency of CLP in the bone marrow cells (*Ythdc1*$^{+/+}$, n=7; *Ythdc1*$^{\Delta/\Delta}$, n = 6).

H. Absolute number of LT-HSC, ST-HSC and MPP in the bone marrow cells (*Ythdc1*$^{+/+}$, n=7; *Ythdc1*$^{\Delta/\Delta}$, n = 6).

I. Absolute number of CMP, MEP and GMP in the bone marrow cells (*Ythdc1*$^{+/+}$, n=7; *Ythdc1*$^{\Delta/\Delta}$, n = 6).

J. Absolute number of CLP in the bone marrow cells (*Ythdc1*$^{+/+}$, n=7; *Ythdc1*$^{\Delta/\Delta}$, n = 6).

Data represent the mean ± SD from n (described above) experiments. **, P<0.01; ***, P<0.001; ****, P<0.0001.

Figure S2. YTHDC1 are required to sustain HSCs during ageing

A. FACS analysis of YTHDC1 protein level in LT-HSC from aged and young mice (n = 4 mice per group).

B. Relative mean fluorescence intensity (MFI) of YTHDC1 in figure S2A (n = 4 mice per genotype).

Data represent the mean ± SD from n (described above) independent experiments. *, P<0.05.

Figure S3. YTHDC1 and METTL3 double knock-out mice displays dissimilar phenotype with METTL3 knockout mice

A. The number of bone marrow cells in wild-type (WT), $Ythdc1^{\Delta/\Delta}$, $Mettl3^{\Delta/\Delta}$ and $Ythdc1^{\Delta/\Delta}Mettl3^{\Delta/\Delta}$ (DKO) mice (n = 6 mice per genotype).

B. Frequency of ST-HSCs, MPP2, MPP3 and MPP4 in the bone marrow cells (n = 6 mice per group).

C. Absolute number of LT-HSCs, ST-HSCs, MPP2, MPP3 and MPP4 in the bone marrow cells (n = 6 mice per group).

Data represent the mean ± SD from n (described above) independent experiments. *, P<0.05; **, P<0.01; ***, P<0.001; ****, P<0.0001; ns, not significant.

Figure S4. Loss of YTHDC1 impairs HSC stemness and affects HSC apoptosis

A. Volcano plot of the transcriptional signature of HSCs in *Ythdc1* $^{\Delta/\Delta}$ relative to *Ythdc1* $^{+/+}$ group.

B. The expression of HSC stemness genes in *Ythdc1* $^{\Delta/\Delta}$ HSC and *Ythdc1* $^{+/+}$ HSC.

C. GSEA analysis of the transcriptional signature of HSC fingerprint in *Ythdc1*$^{\Delta/\Delta}$ relative to *Ythdc1*$^{+/+}$ group.

D. GSEA analysis of the transcriptional signature of WNT signaling pathway in *Ythdc1*$^{\Delta/\Delta}$ relative to *Ythdc1*$^{+/+}$ HSC group.

E. Frequency of apoptotic cells (ANNEXIN V+) in the BM cells from *Ythdc1*$^{+/+}$ and *Ythdc1*$^{\Delta/\Delta}$ mice after in vitro culture (n = 4).

Data represent the mean ± SD from n (described above) dependent experiments. **, P<0.01.

Fig S5. YTHDC1-mediated microRNA processing in HSC is essential for HSC survival and maintenance

A. Schematic representation of the lentivirus vectors used to co-overexpress *miR-99b*, *miR-let-7e* and *miR-125a* (OE-3-miR).

B. Capacity of HP1BP3 and DROSHA interaction reduced in the sorted *Ythdc1*$^{\Delta/\Delta}$ compared with that in *Ythdc1*$^{+/+}$ HSPCs (Lin^{-} c-Kit^{+}).
