# Supplementary figures and images for: YTHDC1-mediated microRNA maturation is essential for hematopoietic stem cells maintenance
Source: Cell Death Discov. 2024 Oct 16;10:439. doi: 10.1038/s41420-024-02203-z (PMC11484846; doi:10.1038/s41420-024-02203-z)

Figure 1B

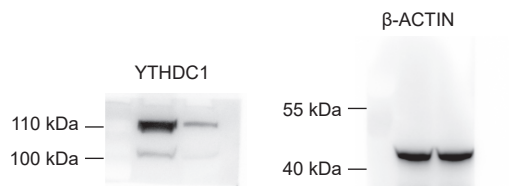

Figure 6E

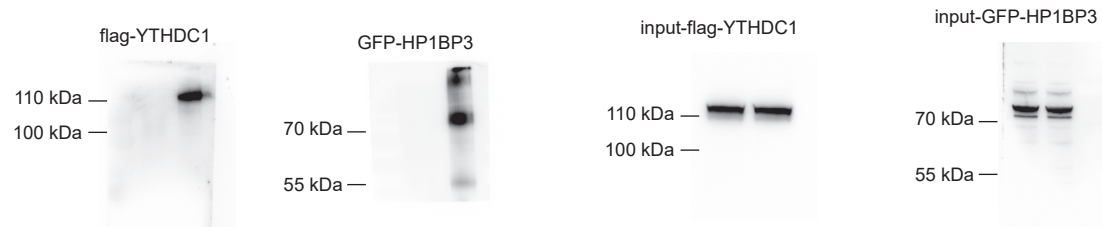

Figure 6F

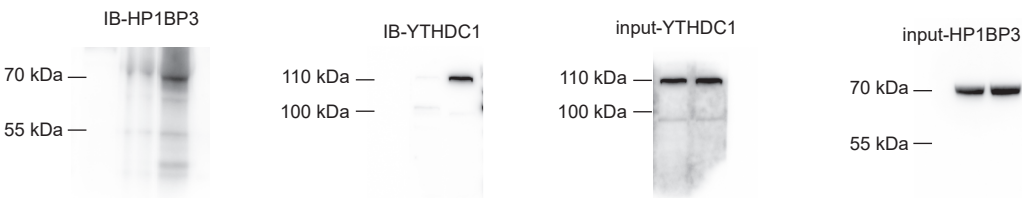

Figure S6B

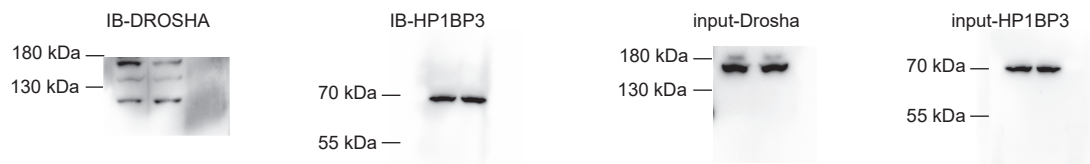

Supplement: Supplementary file 2 — WB origin gel [file 41420_2024_2203_MOESM2_ESM.pdf]
